# Perityphlitic Abscess

**Published:** 1889-06

**Authors:** D. A. Stanton

**Affiliations:** Lexington, N. C.


### PERITYPHLITIC ABSCESS.

BY D. A. STANTON, M. D., LEXINGTON, N. C.

On March 21st, 1889, I was called to see Ambros F., colored, *æt.* 64. I found him suffering great pain in right iliac region. On inquiry the following history was obtained: For three months past he had been constipated, and occasionally suffered lancinating pain in right iliac region, and on the evening before I saw him, was taken with a severe pain in that region, and vomiting. He vomited often during the night, and at the time I saw him he was greatly nauseated, and the pain was so severe that seven grains of opium was required in so many hours to give him relief. At this visit there was only a slight elevation of temperature and pulse. Some tenderness over Appendix Vermiformis. I diagnosed typhlitis, and prescribed opium sufficient to relieve pain, and warm fomentations over site of tenderness.

At my visit next day, found temperature had risen two degrees, and pulse was 96 per minute. Tenderness on pressure had increased, and dullness on percussion was distinct. The case progressed steadily with the usual train of symptoms, and by the seventh day a well defined abscess could be made out. On the eighth day I contemplated cutting through the abscess and putting in a drainage tube, but failed to get patient's consent, he saying he had as well die with the abscess as a hole in his belly. The symptoms not being urgent, I deferred the operation for the time, and on the ninth day a spontaneous opening was made into the bowel and a large quantity of matter passed per *anum*.

After this evacuation of pus his temperature came down and he was comfortable for three days, passing a small quantity of matter each day. On the fourth day the opening closed, and there was a rapid increase in size of tumor, and the symptoms were soon distressing, temperature and pulse going up rapidly, and surface bathed in cold perspiration. I called Dr. Payne, Jr., and after persuading my patient for a time, we got his consent to aspirate. About two ounces of offensive pus was drawn off. In a few hours his temperature was normal and pain all gone. Second day after aspirating, a quantity of matter was passed from bowels. Third day matter was discharged. Fourth day two actions from bowels, each containing pus, and abscess very much reduced. Fifth day only small induration over site of abscess, pain all gone and a very small quantity of matter in evacuation, temperature normal, pulse 72. Gave my patient muriated tincture of iron and quinine, and temporarily dismissed him. Saw him two days later, still improving, appetite good, his condition in every respect favorable, and he was dismissed, able to sit up.

The termination of this case was satisfactory without any other operative interference than the aspirating. But did I do for my patient what modern surgery teaches to be the safest for a case of perityphlitic abscess? I think not. Sands believes in waiting unless there are signs of perforation. Wyeth operates as soon as there are evidences of collection of pus, ascertained by exploring with a small aspirating needle. The latter proceeding seems the most rational surgery, and accords with that time honored law, "Where pus is found let it out."

To wait for symptoms pointing to perforation after we are satisfied that there is a collection of pus, is undoubtedly subjecting our patient to an unnecessary risk. 'Tis true, just where the abscesses are situated is a question *sub judice*, but it is generally conceded that the appendix vermiformis is at fault, and when we remember that the appendix vermiformis is sometimes covered by peritoneum forming a mesentery for it, and the fact that the presence of any foreign substance in the peritoneal cavity is followed by peritonitis, which is often, if not invariably, fatal, we are not justified in exposing our patient to the risk of an abscess emptying its contents into that sensitive cavity. Where such a degree of inflammation has existed for days as is incident to the formation of an abscess, there can be no doubt of the adhesions being sufficient to preclude all danger of an escape of matter into the cavity by opening its walls.

If the abscess is extra-peritoneal there can be no possible danger, and to lay it open and wash it out with carbolic acid solution would certainly be preferable to waiting for nature to make an opening, and if it should be intra-peritoneal, in justice to our patient we ought to make a way for it to escape externally, when there are chances, if we procrastinate, for it to open into the cavity and destroy our patient before an operation could be performed.

It is not modern surgery to wait and subject our patient to a laparotomy and the chance of a peritonitis, when the simple operation would have prevented the latter, to say nothing of the dangers of delay.

